# A Diagnostic Dilemma in Fine-Needle Aspiration Cytology: Spindle Cell/Pleomorphic Lipoma

**DOI:** 10.7759/cureus.20919

**Published:** 2022-01-04

**Authors:** Neetu Bala, Neelam Gupta, Mandeep Sachdeva, Yadvinder Singh, Mahendra Kumar

**Affiliations:** 1 Pathology, Maharishi Markandeshwar University, Solan, IND; 2 Hospital Adminstration, Postgraduate Institute of Medical Education and Research, Chandigarh, IND; 3 Nursing, Postgraduate Institute of Medical Education and Research, Chandigarh, IND

**Keywords:** floret-like giant cell, swelling, molecular abnormalities, multinucleated, pleomorphic lipoma

## Abstract

Pleomorphic lipoma is an uncommon, pseudosarcomatous lesion. It is characterized by the pleomorphic appearance on cytology and histology, follows a benign course, with a low rate of recurrence after complete excision, and has no risk of metastasis. Here, we describe a case of pleomorphic lipoma/spindle cell lipoma in a 41-year-old man who presented with a slow-growing mass on the inner aspect of the left thigh. On fine-needle aspiration cytology, it was reported as a cellular nerve sheath tumour followed by a wide excision sample sent for histopathological examination, which revealed spindle cells exhibiting pleomorphism with mature adipocytic cells and multinucleated floret cells in a myxoid background. It was reported as pleomorphic lipoma on histological examination.

## Introduction

Pleomorphic lipoma is an uncommon variant of lipoma, which typically occurs in middle-aged men. It presents as a well-circumscribed subcutaneous mass most commonly found in the posterior neck, shoulder, and back region. In 1975, first of all, Enzinger and Harvey described this distinct subtype of lipoma as a spindle cell lipoma [1]. Approximately a decade later, Shmookler and Enzinger described another variant of lipoma characterized by the presence of multinucleated floret-like giant cells and they introduced the term pleomorphic lipoma [2]. It is a pseudosarcomatous lesion of the soft tissue and other sites. Since the initial identification of these two entities, it was observed that both the tumours share similar clinicopathological features and molecular abnormalities, specifically the loss of 16q and 13q. Correct diagnosis of such lesions is of key importance because of their good prognosis and prevention of overtreatment in the form of extensive surgery and other therapies [3].

## Case presentation

**Figure 1:**
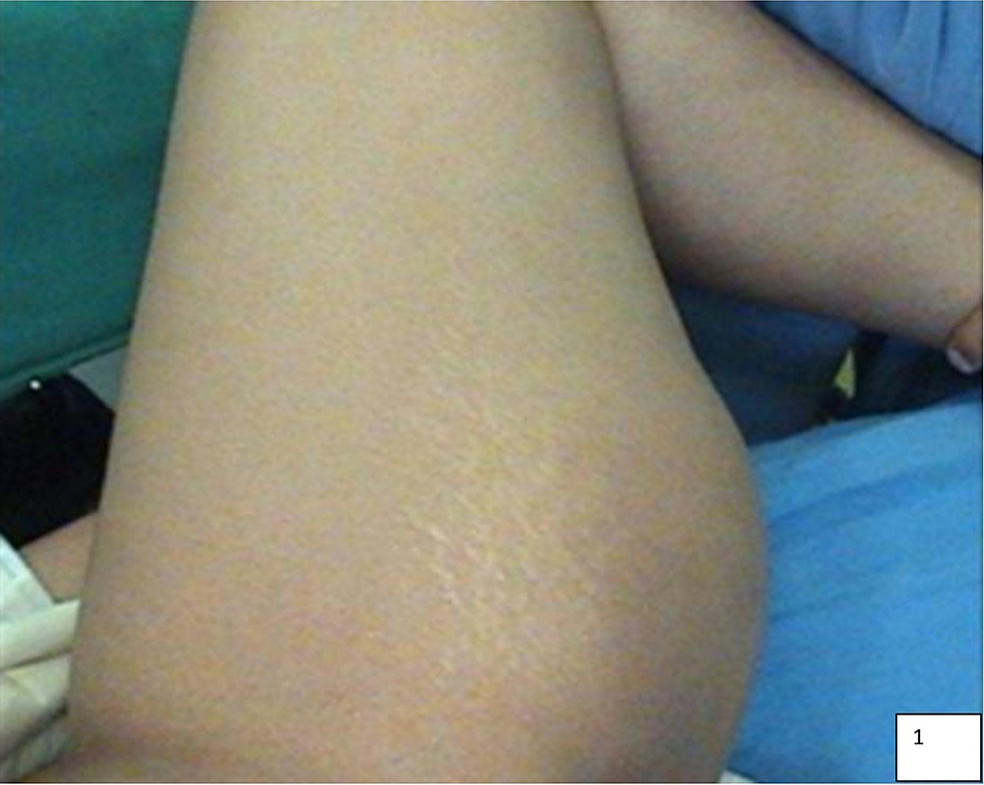
Clinical presentation with left thigh mass.

A 41-year-old male presented to the surgical outpatient department with a slow-growing swelling (5 x 2.5 x 2 cm) for 1.5 years in the medial aspect of the left thigh (Figure 1). The swelling was mobile, soft in consistency, lobulated, and had a smooth surface. It was gradually increasing in size and was associated with occasional pain on pressure, which was aggravated on walking and relieved by lying down. On physical examination, the possibility of soft tissue neoplasm was kept and the patient was sent for fine-needle aspiration cytology (FNAC) and other preliminary investigations. Routine investigations such as complete blood count (CBC), urine routine, and X-ray of the left leg were done, which were within normal limits.

**Figure 2:**
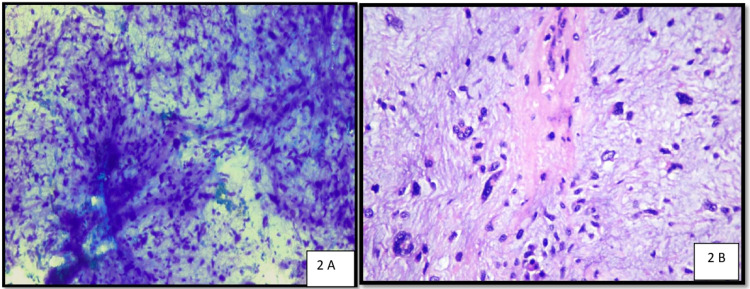
Cytology and histopathology images. A: Cytomorphological picture revealing pleomorphic spindle cells (Giemsa stain, 400x). B: Histopathological picture revealing multinucleate floret giant cells, pleomorphic spindle cells, and extensive myxoid stroma (hematoxylin & eosin stain, 400X).

FNAC was performed and smears revealed many cellular fragments associated with fibrillary intercellular material, pleomorphic cells having indistinct margins, large irregular nuclei, and smudged chromatin present along with some binucleated and multinucleated cells in a myxoid background. Cytological diagnosis of cellular peripheral nerve sheath tumour with suspicion of malignant peripheral nerve sheath tumour (MPNST) was rendered (Figure 2A). The patient also underwent an MRI of the left thigh, which reported a peripheral nerve sheath tumour with no deep extension. Wide excision of the tumour was done and sent for histopathological examination. On gross examination, it was a well-encapsulated and well-circumscribed tumour measuring 4.5 x 2.5 x 2 cm with the lobulated and congested outer surface. The cut surface was yellow in colour with focal myxoid areas. Histopathological analysis revealed a well-encapsulated subcutaneous tumour comprising of round to oval to spindle cells in a myxoid matrix along with foci of mature adipocytic cells. Tumour cells were highly pleomorphic with coarse chromatin, eosinophilic to vacuolated cytoplasm, and a few multinucleated floret cells with indented nuclei. Scanty degenerated fat lobules were also seen and a final impression of pleomorphic lipoma with extensive myxoid change was rendered (Figure 2B). The patient was discharged after excision and was followed up.

## Discussion

Pleomorphic lipoma is an uncommon benign tumour that mainly involves subcutaneous tissue but can arise in the dermis and epidermis also. In 80% of the cases, it is characteristically seen over the neck, shoulder, and back but in 20% of cases, it involves other sites like the pharynx, oral cavity, parotid gland, and female genital tract. In general, a pleomorphic lipoma is encountered in men and the predominant age group is 45-60 years [4-5].

FNAC is a well-known diagnostic technique in experienced hands for the diagnosis of several subcutaneous lesions, including adipocytic tumours. Akerman and Rydholm reported >94% accuracy in lipomatous tumours (both benign and malignant) diagnosed by FNAC [6]. However, spindle cell/pleomorphic lipoma can mimic malignant soft tissue tumours in cytologic aspirates. To the best of our knowledge, a few case reports have described the cytological features of pleomorphic lipoma and out of them, only two cases were diagnosed correctly on FNAC, and the rest of all cases were diagnosed as anaplastic carcinoma/soft tissue sarcoma [6]. The present case was also considered suspicious of MPNST on cytology. But on histopathological examination, the diagnosis of pleomorphic lipoma was given.

Morphologically, it resembles the usual type of lipoma on gross examination but some tumours may have a myxoid change, plexiform architecture, and nodular appearance. Pleomorphic lipoma is a variant of spindle cell lipoma and both these entities share similar histological and immunohistochemical features [7]. On histopathological examination, it can vary in lipomatous and spindle cell content. These varieties may have a complete absence of lipoblasts or atypical lipogenic cells. Similarly, in our case, both spindle and pleomorphic cells were present. Spindle cells exhibited pleomorphism but there was no evidence of lipoblasts in any of the sections examined. Lipoma and its variants can be treated through minimally invasive procedures (local excision), even non-invasive procedures like intralesional steroid injections or liposuctions are also performed nowadays. MPNST is an aggressive tumour and needs wide resection with adjuvant radiotherapy. The other differential diagnoses which need to be ruled out in a case of spindle cell/pleomorphic lipoma with a prominent myxoid component are myxoma, myxoid neurofibroma, and myxoid liposarcoma [8].

## Conclusions

The current case highlights the fact that pleomorphic lipoma is an uncommon, benign adipocytic tumour that can resemble a variety of malignant soft-tissue tumours. Therefore, careful examination of characteristic histopathological features is essential to reach the correct diagnosis and to avoid overtreatment and other therapies.
